# Oxidative Stress Relevance in the Pathogenesis of the Rheumatoid Arthritis: A Systematic Review

**DOI:** 10.1155/2016/6097417

**Published:** 2016-05-31

**Authors:** Celia María Quiñonez-Flores, Susana Aideé González-Chávez, Danyella Del Río Nájera, César Pacheco-Tena

**Affiliations:** ^1^Facultad de Ciencias de la Cultura Física, Universidad Autónoma de Chihuahua, Circuito No. 1, Nuevo Campus Universitario, Apartado Postal 1552, 31240 Chihuahua, CHIH, Mexico; ^2^Facultad de Medicina y Ciencias Biomédicas, Universidad Autónoma de Chihuahua, Circuito No. 1, Nuevo Campus Universitario, Apartado Postal 1552, 31240 Chihuahua, CHIH, Mexico

## Abstract

Rheumatoid arthritis (RA) is an autoimmune inflammatory disease whose pathogenic mechanisms remain to be elucidated. The oxidative stress and antioxidants play an important role in the disease process of RA. The study of oxidants and antioxidants biomarkers in RA patients could improve our understanding of disease pathogenesis; likely determining the oxidative stress levels in these patients could prove helpful in assessing disease activity and might also have prognostic implications. To date, the usefulness of oxidative stress biomarkers in RA patients is unclear and the evidence supporting them is heterogeneous. In order to resume and update the information in the status of oxidants and antioxidants and their connection as biomarkers in RA, we performed a systematic literature search in the PubMed database, including clinical trials published in the last five years using the word combination “rheumatoid arthritis oxidative stress”. In conclusion, this review supports the fact that the oxidative stress is an active process in RA pathogenesis interrelated to other better known pathogenic elements. However, some controversial results preclude a definite conclusion.

## 1. Introduction

Rheumatoid arthritis (RA) is an autoimmune disease affecting diarthrodial joints. It is characterized by erosive synovitis, which causes cartilage and bone destruction and systemic complications including cardiovascular, pulmonary, psychological, and other skeletal disorders [1]. Several autoantibodies have been associated with RA such as rheumatoid factor (RF) and anti-citrullinated protein antibodies (ACPA). RA significantly decreases patients' functional capacity, increases the morbidity and mortality rates, and results in significant costs for the health and social care systems [2]. The prevalence of RA is 1% of the worldwide population and women are more affected. Although the onset is more frequent during the fourth and fifth decades of life, RA can occur at any age [3]. The etiology and pathogenesis of this disease remain unresolved. It is thought that interactions among various factors, including genetic and environmental factors, lead to an inappropriate immunomodulation and result in an inflammatory process resulting in the damage of synovial structures [1]. Regardless of the exact trigger, the reactive oxygen species (ROS) have been implicated to play an important role in this process [4].

**Figure 1:**
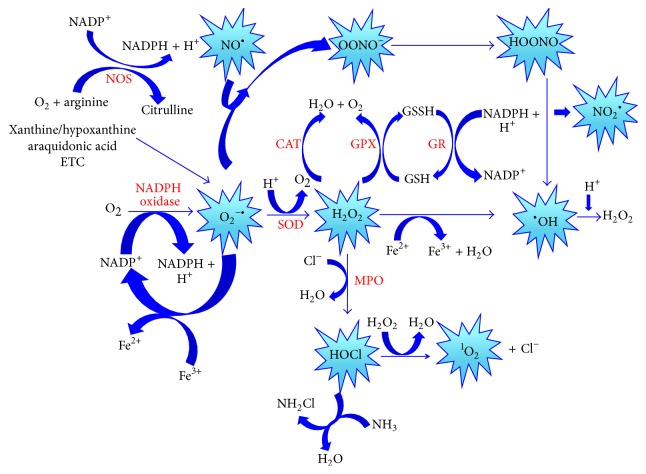
Generation of oxygen and nitrogen reactive species (ROS and RNS). CAT: catalase, ETC: electron transport chain, H_2_O: water, H_2_O_2_: hydrogen peroxide, HOCl: hypochlorous acid, HOONO: peroxynitrous acid, GPx: glutathione peroxidase, GR: glutathione reductase, GSH: reduced glutathione, GSSH: oxidized glutathione, MPO: myeloperoxidase, NADPH: reduced nicotinamide adenine dinucleotide phosphate, NOS: nitric oxide synthase, NH_2_Cl: chloramine, NH_3_: ammonia, NO^•^: nitric oxide, NO_2_^•^: nitrogen dioxide, O_2_: oxygen, ^1^O_2_: singlet oxygen, O_2_^−•^: superoxide anion, ^•^OH: hydroxyl radical, OONO^−^: peroxynitrite, and SOD: superoxide dismutase.

ROS are the most important class of radicals generated in living systems. They are oxygen-derived radicals and include the superoxide radical (O_2_^−•^), peroxyl radical (ROO^•^), perhydroxyl radical (HO_2_^•^) and hydroxyl radical (^•^OH), and non-free radical species such as hydrogen peroxide (H_2_O_2_) and singlet oxygen (^1^O_2_) that are easily converted into free radicals. Nitric oxide (NO^•^), nitrogen dioxide (NO_2_^•^), and peroxynitrite (OONO^−^) represent the most important Reactive Nitrogen Species (RNS) [5]. These chemical species contain one or more unpaired electrons in the outermost orbital shell and are called free radicals [6]. They are unstable, highly reactive and short-lived. Free radicals can abstract electrons from other compounds to attain stability; thus the attacked molecule loses its electron and becomes a free radical itself, beginning a chain reaction cascade [5]. The ROS and RNS generation processes are represented in Figure 1.

Under physiological conditions, ROS are required to maintain the cell redox state and play a role in cell signaling, differentiation, proliferation, growth, apoptosis, cytoskeletal regulation, and phagocytosis. However if the concentrations of ROS are increased beyond physiological conditions they can damage cellular components, such as the lipids in the cell membranes, and also proteins and nucleic acids. If a given condition induces an imbalance between oxidants and antioxidants, where oxidants are favored, a disruption of redox signaling is produced, and a control and/or molecular damage occurs. This cellular state termed oxidative stress [4] can result from an excess of oxidants, antioxidants deficiency, or both conditions [7].

The damaging effect of free radicals is counteracted by the action of antioxidants. An antioxidant is any substance or compound capable to scavenge free radicals or inhibiting the oxidation process in the cell [8]. Enzymatic antioxidant response is carried out by superoxide dismutase (SOD), catalase (CAT), and glutathione (GSH) related enzymes (glutathione peroxidase [GPx], glutathione reductase [GR], and thioredoxin reductase). Furthermore, the nonenzymatic antioxidant response includes the action of vitamins (A, C, and E), *β*-carotene, antioxidant minerals (copper, ferritin, zinc, manganese, and selenium) and L-*γ*-glutamyl-L-cysteinylglycine (GSH), which is the most important nonenzymatic antioxidant defense [4, 9].

RA is one of the conditions that induce oxidative stress. A fivefold increase in mitochondrial ROS production in whole blood and monocytes of RA patients—compared with healthy subjects—suggests that oxidative stress is a pathogenic hallmark in RA. Free radicals are indirectly implicated in joint damage because they also play an important role as secondary messengers in inflammatory and immunological cellular response in RA. T-cell exposure to increased oxidative stress becomes refractory to several stimuli including those for growth and death and may perpetuate the abnormal immune response [10]. On the other hand, free radicals can degrade directly the joint cartilage, attacking its proteoglycan and inhibiting its synthesis [11]. Oxidative damage of hyaluronic acid and lipoperoxidation products and oxidation of low-density lipoproteins and carbonyl increment resulting from protein oxidation have also been demonstrated in RA as well as DNA damage. ROS-induced genotoxic events have also been linked to mutation of p53 in RA-derived fibroblast-like synoviocytes [9]. Furthermore, it has been suggested that antioxidants systems, either enzymatic or not, are impaired in RA. Low levels of GSH [12], tocopherols, *β*-carotene, and retinols and low activities of GR and SOD have been associated [13].

The chronic oxidative stress in the RA synovium has been explained by the elevated intra-articular pressure in RA joints, which increases ROS production in the cellular oxidative phosphorylation and induces repetitive cycles of hypoxia/reoxygenation. The hypoxia is an event observed in RA joints whose origin has been explained to be a consequence of the rapid cellular proliferation induced by the inflammatory response; however, according Jeon et al. [14], the hypoxia precedes inflammation at least in an animal arthritis model. From the “Danger Model” point of view, in which the synoviocyte is an impaired cell, this sequence of events could be happening in the human disease [15]. Activated phagocytic cells can also enhance this oxidative stress during oxidative burst. Environmental factors such smoking, drugs, and ultraviolet light may also play a role.

The association between oxidative stress and RA has been explored using various oxidant or antioxidant biomarkers. These biomarkers include lipids, proteins, and DNA oxidation markers and also levels of enzymatic activities, antioxidants agents, and even the direct measurement of free radicals. The aim of this review is to resume and update the available evidence in regard to the potential role of oxidants and antioxidants in RA patients and the findings related to these biomarkers in RA. A systematic literature search was performed including studies published in the last 5 years. The findings are presented in a comparative way between studies included.

## 2. Methods

A systematic literature search was performed including studies published between May 2010 and May 2015. These studies assessed oxidant and/or antioxidant biomarkers in RA patients. The search was conducted in the PubMed database. The word combination used for the search was “*rheumatoid arthritis oxidative stress*”. The article selection was performed using the inclusion and exclusion criteria described below.

### 2.1. Selection Criteria

This review included original articles published in English within the last 5 years. The studies were clinical trials, which assessed oxidant and/or antioxidant biomarkers in RA patients. Even if the main purpose of any of the selected articles was to compare different diseases, we selected only the results obtained from RA. Studies with experimental interventions and those with oxidative stress induced by causes other than RA (periodontitis, smoking, nutritional status, and genetic polymorphisms in RA) were excluded.

### 2.2. Methodological Quality

We assessed the methodological quality of the included studies with the Newcastle-Ottawa Quality Assessment Scale (NOS). Every study received a score consisting in a number of stars. The NOS include three domains: (a) selection (maximum 4 stars), (b) comparability (maximum 2 stars), and (c) exposure (maximum 3 stars). The highest score possible was 9 stars. Studies with scores of 6 stars or above were considered to be of moderate to good study quality. The score was not an exclusion criterion. The quality of the selected articles was assessed by one reviewer and checked by a second reviewer.

### 2.3. Analysis of Information

We selected the following information from every included article: age, female/male ratio, sample size, disease activity score (DAS-28), disease duration, type of biological sample, oxidant and antioxidant biomarkers levels, and the findings related to these biomarkers. This information was organized in comparative tables.

## 3. Results

### 3.1. Selected Articles

**Figure 2:**
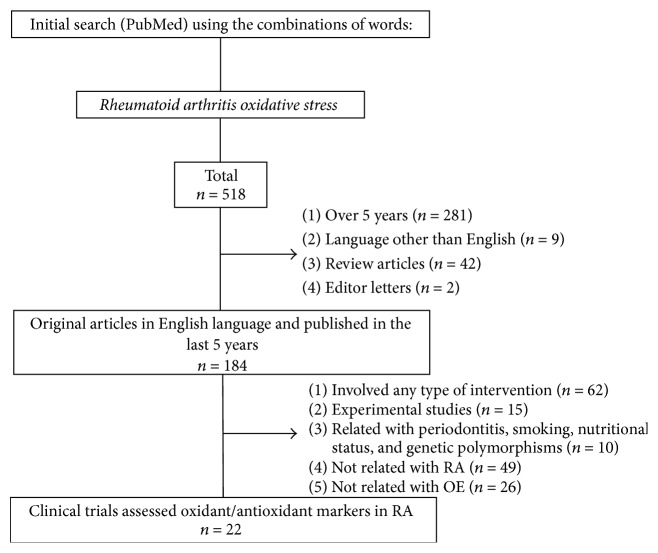
Flow chart of study selection.

The process of article selection is described in Figure 2. From 518 articles, a total of 22 studies fulfilling our inclusion criteria and not the exclusion criteria were selected for this review. Almost all included studies had moderate or high quality. Eight studies were given 8 stars, 9 studies were given 7 stars, 3 studies were given 6 stars, and 1 study was given 4 stars. Datta et al.'s [16] study was included in this review; however this article was not evaluated under NOS criteria due it being not a case-control study. We did not find a published systematic review that was related to the oxidative stress in RA in the last 5 years.

### 3.2. Study Characteristics

The characteristics of the included studies are summarized in Table 1. The number of participating patients ranged from 20 to 1720 (cases groups) and 10 to 120 (control group) among studies. The ratio of women to men was higher in both cases and control groups of included studies, with the exception of the study of Nakajima et al. [17]. In some studies [16, 18–24] the gender information was not fully detailed. The age mean ranged from 24.2 to 63.4 years. Sixteen articles reported the DAS-28 score and/or the disease duration. The mean DAS-28 score ranged from 2.1 to 5.7 and disease duration from 11 months to 25 years. In general, the studies included healthy individuals as a control group, except for the studies reported by Nakajima et al. [17], Ediz et al. [25], and Nzeusseu Toukap et al. [19], in which Diabetes Mellitus (DM), RA anti-ACPA (−), and Osteoarthritis (OA) were included, respectively. Thiele et al. performed one part of their work with healthy individuals and the other in patients with OA as controls. Only the study from Datta et al. [16] did not include a control group.

In regard to the country of the populations included, 5 studies were conducted in India, 4 in Turkey, 2 in Poland, 1 in New Zealand, 1 in Iran, 2 in USA, 1 in Serbia, 1 in Egypt, 1 in Mexico, 1 in Australia, 1 in Japan, 1 in Belgium, and 1 in China.

### 3.3. Oxidant and Antioxidant Markers Measured in RA

Thirty different oxidant and/or antioxidant markers were analyzed among the selected studies. They were classified in seven groups: (1) lipid peroxidation (4 markers: malondialdehyde [MDA], thiobarbituric acid reactive substances [TBARS], isoprostane [F2-I], and, malondialdehyde-acetaldehyde [MAA], adducts), (2) protein oxidation (4 markers: protein carbonyls [PC], 3-chlorotyrosine [CT], advanced oxidation of protein products [AOPP], and nitrosothiols [RSNO]), (3) DNA damage (2 markers: micronucleus [MN] and DNA stand breaks [DNA sb]), (4) urate oxidation (1 marker: allantoin [ALLA]), (5) enzymatic activity (7 markers: CAT, SOD, GR, GPx, myeloperoxidase [MPO], NADPH oxidase [NADPH ox], and arylesterase [AE]), (6) antioxidants (6 markers: GSH, oxidized glutathione [GSSG], *β*-carotene [*β*C], vitamin E [VE], SH group, and total antioxidant capacity [Anti-Cap]), and (7) free radical/anions (6 markers: total ROS, reactive oxygen metabolites (ROM), H_2_O_2_, O_2_^−•^, ^•^OH, and NO^•^) (Table 2). The oxidation of lipids biomarkers was extensively studied (16/22 articles), followed by enzymatic activity (15/22), antioxidants (9/22), protein oxidation (5/22), free radical and anions (4/22), DNA damage (1/22), and uric acid oxidation (1/22). The biological samples used in the studies were blood (whole blood, serum, plasma, erythrocytes, and lymphocytes), synovial fluid, synovial tissue, and urine. The blood sample was the most utilized.

### 3.4. Lipid Oxidation

Sixteen of the 22 studies assessed lipid oxidation biomarkers (MDA, TBARS, F2-I, and MAA adducts). Ten of them measured MDA levels. Most of them observed a statistically significant increase in MDA blood levels in RA patients. A significant difference in MDA blood concentration between RA and control patients was not reported by Jacobson et al. [26] and Ediz et al. [25]; however, Ediz et al., who also measured MDA from RA synovial fluid, reported an MDA increase in this sample. The MDA levels in synovial fluid reported by Datta et al. [16] correlated with levels of both ROS and ^•^OH radicals and interestingly with the DAS-28 score suggesting an association with disease activity. No study included in this review reported a decrease in MDA levels in patients with RA compared to controls. The blood TBARS levels were measured in 3 studies [21, 27, 28], which reported a significant increase of these biomarkers in RA.

The F2-I levels were reported in two studies. A significantly higher F2-I excretion in patients with RA than control subjects was found by Rho et al. [23]. This biomarker was associated with a loss of protective effect of HDL cholesterol against coronary calcification. Additionally, plasma levels of F2-I were higher in RA patients compared to controls in the study reported by Kwaśny-Krochin et al. [29]. A positive association between plasma asymmetric dimethylarginine (ADMA) and F2-I and C-Reactive Protein (CRP) concentration in RA samples was reported.

The MAA adducts expression in RA synovial tissue was evaluated only in one study [18] and it was increased in patients with RA if compared to OA. Interestingly, MAA adducts colocalized with citrullinated proteins. Furthermore, increased levels of anti-MAA antibody also correlated to seropositivity for ACPA and RF, suggesting a potential pathogenic role.

### 3.5. Protein Oxidation

Protein oxidation was evaluated through different biomarkers (PC, RSNO, AOPP, and CT) in 5 studies [16, 19, 20, 24, 27]. The grade of protein carbonylation was higher in plasma from RA patients if compared with healthy controls in 3 studies [20, 24, 27]. Datta et al. [16] found AOPP protein carbonylation, RSN, and PC present in the synovial fluid from RA patients and also a positive correlation between these biomarkers and DAS-28 score. The level of CT in synovial fluid was also higher in RA patients than in controls [19].

### 3.6. DNA Oxidation

Only one study assessed the DNA damage by MN and DNAsb (comet assay). Karaman et al. [30] reported DNA damage in RA lymphocytes, in parallel with an increase of MDA levels and decrease in SOD and GPx activity. These results suggested an increase on oxidative stress in RA, a situation that may impair genetic stability.

### 3.7. Acid Uric Oxidation

The ALLA plasma concentration as a measure of oxidation of urate was assessed by Stamp et al. [20] and it was higher in RA patients compared with controls. However no correlation with other markers such as MPO or protein carbonyls was found.

### 3.8. Enzymatic Activity

Enzymatic activity was evaluated in 15 of the included studies (GPx, SOD, CAT, GR, AE, NADPH ox, and MPO); the results were heterogeneous. The enzyme most commonly assessed was GPx (8 studies); it was measured in red blood cells [10, 21, 31] or in plasma/serum [25–27, 30, 32]. A GPx decreased activity was reported in RA patients in 3 studies [10, 30, 31]; however 2 studies conversely reported an increase on GPx activity in RA patients [26, 27] and 3 more studies did not report any differences between cases and controls in whole blood, serum, synovial fluid, plasma, and erythrocytes [21, 25, 32]. Therefore rendering a conclusion seems complicated at this point.

The SOD activity was also evaluated and gave conflicting results. In 5 studies, the SOD activity from RA patients was found lower than in controls in plasma and erythrocytes [21, 22, 30–32]. Moreover, a higher activity of SOD in RA patients was found in 2 studies [27, 28].

CAT activity was evaluated in 5 studies. Four of these studies [21, 25, 28, 31] did not report any differences between RA patients and controls, whereas the study conducted by Shah et al. [32] showed a lower CAT activity in plasma of RA patients than in healthy controls.

Evidence regarding GR, AE, and NADPH ox activity in RA was limited in the included studies [22, 33, 34]. GR activity was found significantly lower in RA cases than controls [22, 34]; however, AE activity did not show differences between cases and controls [34]. NADPH ox activity was 1.75-fold higher in RA plasma than healthy individuals. Furthermore, the NADPH activity was significantly higher in RA synovial fluid than in RA peripheral blood [33].

MPO activity was reported in 4 of the included studies. A decrease in the MPO activity in RA plasma was reported by Stamp et al. [20]; however, the activity in synovial fibroblast was found to have increased. Moreover, a significant increase in MPO activity in synovial fluid was reported [19, 25], but not in whole blood and serum [25]. On the other hand, an increase in serum MPO concentration in RA was found in 2 studies [20, 35].

### 3.9. Nonenzymatic Antioxidants

Nine studies assessed different antioxidants molecules. GSH concentrations were measured in 6 studies. With respect to control group, this biomarker was found diminished in 3 studies [10, 21, 32], increased in 2 [27, 31], and unchanged in 1 [28] in RA patients. GSSG levels were evaluated only in one study [27], with no differences between RA patients and the control group. However, the GSH/GSSG ratio was significantly elevated in control group compared to RA patients suggesting that high levels of GSH are associated to the higher GR activity (reduction of GSSH to GSH) in RA compared with controls. *β*C and VE antioxidants were determined in one study [34] and were lower in RA patients than controls. Likewise, SH groups were assessed and reported decreased in RA samples for 2 studies [21, 24]. Total antioxidant capacity assay was used in one study in which no differences were found between RA and control group [26].

### 3.10. Free Radicals/Anions

Total ROS, ROM, H_2_O_2_, O_2_^−•^, ^•^OH, and NO^•^ were used as oxidative stress biomarkers in 4 studies [16, 17, 28, 33]. All these biomarkers were found elevated in RA patients suggesting an active oxidative process. Only for NO^•^ contrasting findings were reported among studies. No differences were found in NO^•^ plasma levels between RA and control group [28]; however, NO^•^ in monocytes from blood of RA patients was higher than control group [33].

## 4. Discussion

The aim of this review was to show and update the available evidence in regard to oxidants and antioxidants in RA patients and to highlight the findings related to biomarkers. Several oxidative stress molecules have been explored as potential biomarkers to monitor the disease progression and explore their role in the RA pathogenesis. Therefore, we consider that it was relevant to review the information in the last 5 years in a systematized approach. To our knowledge, no systematic review that analyzes this set of biomarkers has been published within the last 5 years.

**Figure 3:**
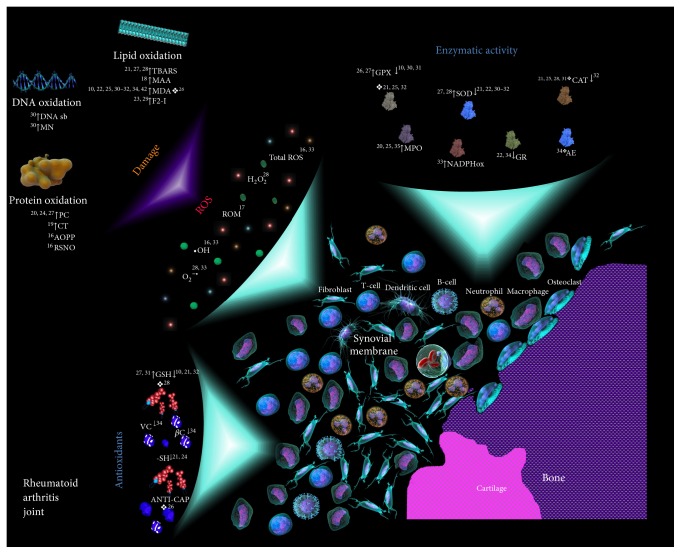
Oxidants/antioxidants biomarkers and oxidative damage found in joints and blood of RA patients. The literature references pertaining to the indicated phenomena are provided in the scheme. Under oxidative stress conditions, the joints and blood of patients with RA show high concentrations of free radicals, mainly ROS (gray), which induce DNA, proteins, and lipids damage through different mechanisms. The nonenzymatic antioxidant response (blue square), in general, is diminished. The enzymatic activity (including enzymatic antioxidant response) shows variability. AE: arylesterase, ANTI-CAP: total antioxidant capacity, AOPP: advanced oxidation protein products, CAT: catalase, CT: 3-chlorotyrosine, DNA sb: DNA strand breaks, F2-I: F2-isoprostane, GPx: glutathione peroxidase, GR: glutathione reductase, GSH: reduced glutathione, H_2_O_2_: hydrogen peroxide, MAA: malondialdehyde-acetaldehyde, MDA: malondialdehyde, MN: micronucleus, MPO: myeloperoxidase, NADPH ox: reduced nicotinamide adenine dinucleotide phosphate oxidase, O_2_^−•^: superoxide anion, ^•^OH: hydroxyl radical, PC: protein carbonyls, RA: Rheumatoid Arthritis, ROM: reactive oxygen metabolites, ROS: reactive oxygen species, RSNO: S-nitrosothiols, -SH: thiol group, SOD: superoxide dismutase, TBARS: thiobarbituric acid reactive substances, VE: vitamin E, and *β*C: *β*-carotene. ^↑^Significantly elevated levels, ^↓^significantly diminished levels, and ^*❖*^no significantly difference.

In our revision, the information connecting free radical biomarkers with the oxidative damage was very consistent (Figure 3). In all the studies, total ROS, H_2_O_2_, O_2_^−•^, and ^•^OH were higher in RA patients regardless of the sample used. Interestingly, a new method called d-ROM, which measures ROM in blood, was used in one study [17]. ROM were used as an overall oxidative stress parameter because they are relatively more stable in the blood than direct measurement of ROS. In this study, the ROM concentrations were increased in RA samples compared to controls and correlated with DAS-28 scores and CRP. This information suggests that the use of ROM in conjunction with CRP could be a useful biomarker to evaluate the activity of the disease. These results are consistent with the findings reported by Hayashi et al. [36] in a nutritional study (therefore was not included in this review) where higher levels of ROM in blood and saliva from RA patients also correlated with DAS-28 scores.

Other free radicals biomarkers were analyzed in the included studies. Veselinovic et al. [28] reported significantly higher levels of O_2_^−•^ and H_2_O_2_ in RA plasma. The authors suppose that O_2_^−•^ radicals in plasma could be dismutated to produce H_2_O_2_ by an upregulated SOD, but CAT or GSH did not detoxify the H_2_O_2_. In the same study, NO^•^ negatively correlated with GSH, which is a possible compensatory effect of intracellular nonenzymatic antioxidative mechanisms to an increased NO_2_^•^ production. Kundu et al. [33] found that levels of total ROS, O_2_^−•^, and ^•^OH radicals were significantly increased in neutrophils from peripheral blood and synovial infiltrate and also showed a strong positive correlation with both DAS-28 and CRP/ACPA levels. Positive correlations of these free radicals with parameters of disease activity and prognosis suggest that the measurement of these biomarkers in RA patients, combined with current markers, could be useful for monitoring disease activity.

The oxidative damage biomarkers (lipid, proteins, uric acid, and DNA oxidation) were also analyzed, aside from the free radicals. In these studies it was shown that the oxidative damage biomarkers are consistently and significantly higher in RA patients if compared to control individuals; this increment was observed in any sample analyzed (serum, plasma, erythrocytes, urine, synovial fluid, and whole blood). An oxidative stress environment prevails in RA, which results in the oxidation of biomolecules in this disease.

Lipid peroxidation is one of the major consequences of oxidative stress. It alters the fluidity and permeability of cell membranes and impairs the activity of membrane-bound enzymes. Lipid peroxidation leads to the production of conjugated diene hydroperoxides and unstable substances, which disintegrate into various bioactive aldehydes such as MDA, 4-hydroxynonenal (HNE), and TBARS [5]. It is known that MDA and HNE can alter protein structures and render them antigenic [37]. In 4 of the included studies, MDA values positively correlated with DAS-28 score [10, 16, 32, 33]. Moreover, Alver et al. [31] found increased MDA levels in RA serum and erythrocyte in combination with a negative correlation between carbonic anhydrase (CA) II antibody levels and SOD activity. This evidence is consistent with findings in the SOD knockout mice, in which the elevated oxidative stress in erythrocytes causes anti-CA II antibody production [38]. These results suggest that oxidative stress may enhance the antigenic properties of CA II and promote the production of autoantibodies and also indicate that MDA measurement in RA samples could be an effective option to monitoring the disease activity.

Additionally, in the included studies, the anti-MAA antibodies correlated closely with ACPA [18]. Indeed, the presence of ACPA was associated to increased levels of MDA and MPO in RA synovial fluid levels [25]. It is known that MDA can spontaneously break down and form acetaldehyde (AA). Both MDA and AA are highly reactive aldehydes, and together they modify proteins to produce MDA-AA protein adducts called MAA, which are highly immunogenic [39, 40]. Presumably, MDA modification of constitutive proteins into RA-related neoantigens could play an important role in the generation of immune responses in RA. Interestingly, MAA adducts colocalize with citrullinated proteins in the inflamed synovial tissue of RA patients, but not in the synovial tissue of OA patients and, furthermore, the anti-MAA antibody levels are associated with seropositivity for ACPA and RF levels [18]. Positive RA patients represent a subset of RA that is characterized by an aggressive disease, including early and progressive bone erosions [41]. The ACPA are produced at least in part in the synovial structures and can enhance oxidative stress in the joints of ACPA positive RA patients. This increased oxidative activity in synovial fluid may be a factor for accelerated bone erosion seen in ACPA positive RA patients.

The effect of oxidative stress on lipids was analyzed in 3 of the included studies. Kwaśny-Krochin et al. [29] described a positive correlation between ADMA levels and the production of isoprostanes and CRP in RA. Moreover, Mishra et al. [42] found that MDA and CRP correlated positively with cholesterol, and Rho et al. [23] reported that isoprostane excretion and HDL cholesterol concentrations are positively associated with the severity of coronary calcification in patients with RA. It is known that oxidative stress and inflammation might augment dyslipidemia in RA, which are risk factors for cardiovascular disease [42]. Dysregulations of hemostasis and local blood flow in vessels are associated with an altered balance between NO^•^ and O_2_^−•^ in endothelial cells. Indeed, the suppression of endothelial NO^•^ synthase activity has been considered a hallmark of endothelial injury initiating atherosclerosis [43]. Elevated levels of ADMA, an endogenous inhibitor of NOS, can be detected in RA patients regardless of the presence of cardiovascular disease [44]. The activity of dimethylarginine dimethylaminohydrolase, the key enzyme in ADMA degradation, is downregulated by oxidative stress and TNF-alpha [45], which plays a crucial role in RA [46]. This data suggests that the oxidative stress and the inhibition of NOS by ADMA could play an important role in the pathogenesis of vascular injury in RA.

Another way to assess oxidative damage in RA is throughout its effect on proteins. Free radicals can modify both their structure and functions. The presence of elevated protein carbonyls in RA samples, produced either by direct oxidation of certain amino acids, by a secondary reaction with HNE, or by a glycoxidation reaction [47], suggests a strong oxidative stress state since the carbonyl formation requires high levels of oxidative stress which is detectable in the rheumatoid synovium.

Stamp et al. [20] found elevated levels of protein carbonyls and 3-chlorotyrosine along with an increased MPO activity in RA fluid synovial. Since MPO is the unique human enzyme capable of producing 3-chlotyrosine, the detection of this molecule confirms the production of hypochlorous acid in synovial fluid. Furthermore, the association of protein carbonyls with both MPO and 3-clorotyrosine suggests that hypochlorous acid has a main role in the protein oxidation in this site. Additionally, MPO converts LDL into an isoform that promotes foam cell formation within atherosclerotic plaques and promotes a dysfunctional form of HDL [48]. Elevated levels of MPO in RA and its contribution to oxidative stress provide a potential mechanism for the increase in cardiovascular complications observed in RA patients.

Additionally, in one of the included studies, AOPP, protein carbonyls, and RSNO levels were found elevated in RA synovial fluid and correlated positively with DAS-28. Proteins carbonyls also correlated positively with ROS and ^•^OH radical [16]. AOPPs are considered stable oxidation protein biomarkers and include dityrosine-containing and cross-linking protein products mainly formed during oxidative stress by the reaction of plasma albumin with chlorinated oxidants. Moreover,* in vitro* studies demonstrated that AOPP induce inflammatory response in fibroblast-like synoviocytes (FLSs) mediated by NADPH oxidase-dependent of NF-*κ*B activation [49]. Meanwhile, RSNO can disrupt the protein structure and interfere with the catalytic activity of various enzymes. RSNO measure reflects the amount of NO^•^ in adducts with cellular -SH compounds. This data suggests that measurement of biomarkers of protein oxidation could be used to monitor the severity of the disease in RA patients.

The use of antioxidant molecules as indirect biomarkers of oxidative stress was also included in our revision. Of the antioxidants analyzed in the included studies, the *β*-carotene, vitamin E, and SH group were consistently found decreased. However, the GSH levels were discrepant between studies. These results suggest that the antioxidants systems are impaired in RA. In contrast to these findings, Jacobson et al. [26] did not find a difference in total antioxidant capacity between cases and controls. This opposite finding could be explained by the fact that, in some studies, the oxidants and antioxidant systems are evaluated in a partial and independent way. This evaluation could lead to errors in the interpretation of results, since the total oxidative status cannot be demonstrated. The oxidative stress usually is interpreted as the increase in oxidants or the decrease in antioxidants; however, in this interpretation, the fact that the oxidative stress reflects the final effects of the combined action of oxidant and antioxidant systems is not considered. Indeed, there may be individuals with high levels of oxidant molecules but with an efficient antioxidant response, as well as subjects without elevated oxidant concentrations but with a deficient antioxidant response. Therefore, the measurement of total oxidant-antioxidant status is the most valid and reliable way to assess the oxidative stress.

Finally, the activity of antioxidant enzymes (SOD, CAT, GPx, and GR) was evaluated in some of the included studies [10, 21, 25–28, 30–32]. The results from these studies were inconsistent and inconclusive. The antioxidant enzyme activity was found increased, decreased, and even equal in RA patients compared to the control subjects. This high heterogeneity observed among studies makes it difficult to draw clear conclusions. The decrease in antioxidant enzyme activity could be explained by the saturation of the enzymatic antioxidant systems and by the enzymatic inhibition, such as the SOD inhibition by hydrogen peroxide [50]. Particularly, CAT activity was found unchanged; this enzyme does not show significant activity under physiological conditions due to its lower affinity than GPx for H_2_O_2_ but becomes an important enzyme at disease state where concentration of H_2_O_2_ is elevated [51]. Furthermore the use of different methodologies to determine enzyme activity can produce diverse results.

Gathering our results, we can conclude that oxidative stress is a dynamic and complex phenomenon occurring in RA and that is involved in the disease pathogenesis in a complex fashion. Unfortunately, the actual evidence is discrepant in the role of some oxidative stress related molecules. These discrepancies complicate our understanding of the mechanism of oxidative stress implication in RA. The variability and complexity of the regulating mechanisms of oxidative stress in humans, which are associated with genetic, epigenetic, age, gender, and dietary factors, can explain these discrepancies. The results of the present revision suggest the plausibility of several oxidative stress related compounds as potential biomarkers to assess the disease activity and probably prognosis. However, the biomarkers need to be validated in prospective clinical studies. This process implicates the compliance with certain requirements which include (a) a stable product of oxidative stress, not susceptible to artificial induction or loss during storage, (b) that this product can be detectable in the target tissue or a valid surrogate tissue where it causes oxidative modification and damage, (c) that it is present in sufficient and measurable concentrations, (d) that it could be determined by an assay that is specific, sensitive, reproducible, and robust, (e) that this compound should be free of confounding factors from dietary intake, and (f) that it could be measurable within a detection limit of a reliable analytical procedure. Additionally, it is essential to consider relevant clinical factors that could lead to misinterpretation of results such as disease duration, disease activity, its treatment, and even the patient status at the moment of the sample collection are important confounding factors that could affect the interpretation of the assays.

Although our study indicates important aspects of the status of oxidative stress in RA, it is important to highlight some of its limitations. The search strategy used in this review was limited to the findings of the last five years. Also, the search was conducted in a single database using one set of keywords. Expanding the search criteria the number of articles would increase and possibly allow stronger conclusions. A meta-analysis also would be appropriate.

## Figures and Tables

**Table 1:** Demographic and clinical characteristics of RA patients and control groups.

Author and year	Country	Sample size (% women/% men)		Age in years (mean or Min–Max)		DAS-28 (mean)	Duration of disease (mean or Min–Max)
		Cases	Controls	Cases	Controls		
García-González et al., 2015 [27]	Mexico	10 (90/10) A, 19 (84/16) I^*∗*^	41 (90/10)	48 A, 48.5 I	38.0	4.3 A, 2.1 I	7.0 y A, 2.0 y I
Thiele et al., 2015 [18]	USA	1720 (9.1/90.9)	80 (NS)	63.4	NS	3.9	12.4 y
Datta et al., 2014 [16]	India	36 (77.7/22.3)	NS	40	NS	5.6	11 m–24 y
Nakajima et al., 2014 [17]	Japan	152 (67.7/32.3)	80 (42.5/57.5)	63.1	59.2	3.5	14.3 y
Nzeusseu Toukap et al., 2014 [19]	Belgium	33 URA (NS), 33 TRA (NS)^*∗∗*^	39 (NS)	NS	NS	4.8 URA, 4.9 TRA	NS
Veselinovic et al., 2014 [28]	Serbia	52 (63.5/36.5)	30 (63.2/36.8)	52.4	54.2	3.6	5.7 y
Wang et al., 2014 [35]	China	100 (62/38)	50 (68/32)	55.7	52.5	5.3	7.0 y
Jacobson et al., 2012 [26]	Australia	35 (62.9/37.1)	39 (61.5/38.5)	62.9	62.8	NS	NS
Kundu et al., 2012 [33]	India	25 (80/20)	10 (80/20)	40.0	26.5	5.7	11 m–25 y
Kwaśny-Krochin et al., 2012 [29]	Poland	46 (85/15)	50 (86/14)	57.0	56.0	5.2	8.1 y
Mishra et al., 2012 [42]	India	36 (61.1/38.9)	36 (69.4/30.6)	49.7	49.6	NS	NS
Stamp et al., 2012 [20]	New Zealand	77 (71.4/28.6)	120 (NS)	54.7	NS	3.8	NS
Staroń et al., 2012 [21]	Poland	25 (84/16)	35 (NS)	NS	NS	NS	NS
Alver et al., 2011 [31]	Turkey	52 (76.9/23.1)	42 (73.8/26.2)	49.2	48.5	NS	NS
Aryaeian et al., 2011 [34]	Iran	59 (64.4/35.6)	59 (64.4/35.6)	41.9	39.1	NS	8.2 y
Ediz et al., 2011 [25]	Turkey	25 (72/28) ACPA (+)	24 (76/24) ACPA (−)	54.4	56.2	4.1 ACPA (+), 3.4 ACPA (−)	9.6 y ACPA (+), 8.1 y ACPA (−)
Hassan et al., 2011 [10]	Egypt	30 (100)	30 (100)	35.8	32.3	4.0	6.5 y
Karaman et al., 2011 [30]	Turkey	43 (74.4/25.6)	30 (56.7/43.3)	39.8	37.2	NS	4.2 y
Desai et al., 2010 [22]	India	40 (50/50)	40 (NS)	40–60	40–60	NS	NS
Rho et al., 2010 [23]	USA	169 (NS)	92 (NS)	>18	>18	NS	NS
Shah et al., 2011 [32]	India	30 (83.3/16.7)	30 (90/10)	24.2	26.7	4.5	5.0 y
Tetik et al., 2010 [24]	Turkey	20 (NS)	20 (NS)	48	25	NS	11 y

^*∗*^A: active disease patients; I: inactive disease patients.

^*∗∗*^URA: untreated patients; TRA: treated patients.

ACPA: anti-citrullinated protein antibodies, DAS-28: Disease Activity Score, m: months, NS: not specified, y: years.

**Table 2:** Summary of reported oxidant and antioxidant markers in RA patients compared to control group.

Author	Lipid oxidation	Protein oxidation	DNA oxidation	Urate oxidation	Enzymatic activity	Antioxidants	Free radicals/anions	Main findings
García-González et al., 2015 [27]	TBARS: ^↑^p	PC: ^↑^p	NE	NE	SOD: ^↑^p; GPx: ^↑^p	GSH: ^↑^p; GSSG: ^*∗*^p	NE	Oxidative damage was elevated in RA patients. Antioxidants enzyme activities, GSH levels, and GSH/GSSG ratio were higher in RA than in control group; however they were insufficient to prevent oxidative damage.

Thiele et al., 2015 [18]	MAA: ^↑^st	NE	NE	NE	NE	NE	NE	MAA adduct formation is increased in RA and colocalized with citrullinated proteins. MAA antibodies are associated with ACPA production. This suggests that MAA formation may drive tolerance loss to autoantibody formation in RA.

Datta et al., 2014 [16]	MDA: sf^❖^	PC: sf^❖^; AOPP: sf^❖^; RSNO: sf^❖^	NE	NE	NE	NE	Total ROS: sf^❖^; ^∙^OH: sf^❖^	All oxidative damage markers correlated positively with DAS-28; therefore the measurement of oxidative stress could serve as a biomarker for monitoring disease severity in RA.

Nakajima et al., 2014 [17]	NE	NE	NE	NE	NE	NE	ROM: ^↑^s	Serum level of ROM was associated with CRP and DAS-28 suggesting that ROM may be able to be used as a disease marker to evaluate the disease activity.

Nzeusseu Toukap et al., 2014 [19]	NE	CT: ^↑^sf	NE	NE	MPO: ^↑^sf	NE	NE	MPO activity, MPO, and CT levels were significantly higher in synovial fluid of RA patients than OA patients. MPO activity and concentration were correlated with IL-8 and IL-18 in untreated but not in treated RA patients.

Veselinovic et al., 2014 [28]	TBARS: ^↑^p	NE	NE	NE	CAT: ^*∗*^e; SOD: ^↑^e	GSH: ^*∗*^e	H_2_O_2_: ^↑^p; O_2_^−∙^: ^↑^p; NO^∙^: ^*∗*^p	Higher levels of prooxidants in RA compared to control group. Stronger response in samples with higher diseases activity suggests that oxidative stress markers may be useful in evaluating the progression of RA.

Wang et al., 2014 [35]	NE	NE	NE	NE	MPO: ^↑^s^#^	NE	NE	Serum levels of MPO higher in RA than control group. Moderate positive correlations between MPO levels and CRP, DAS-28. These results support a role for MPO in the inflammatory process of RA.

Jacobson et al., 2012 [26]	MDA: ^*∗*^p	NE	NE	NE	GPx: ^↑^p^#^	Anti-Cap: ^*∗*^p	NE	There were no differences between RA cases and control group for oxidative stress and antioxidant capacity; however, GPx level was markedly elevated in RA. GPx levels were not associated with severity disease or CRP.

Kundu et al., 2012 [33]	NE	NE	NE	NE	NADPH ox: ^↑^p, ^✪↑^sf	NE	Total ROS: ^↑^n-b, ^↑✪^n-sf; O_2_^−∙^: ^↑^n-b, ^↑✪^n-sf; ^∙^OH: ^↑^n-b, ^↑✪^n-sf; NO^∙^: ^↑^n-b, ^↑✪^n-sf	ROS generated in both peripheral blood and synovial infiltrate correlated positively with both DAS-28 and CRP/ACPA levels; its measurement can serve as an indirect measure of the degree of inflammation in patients with RA.

Kwaśny-Krochin et al., 2012 [29]	F2-I: ^↑^p	NE	NE	NE	NE	NE	NE	ADMA levels are significantly higher in RA than in control group. Positive associations between plasma ADMA levels and the production of 8-isoprostanes and CRP in RA.

Mishra et al., 2012 [42]	MDA: s^↑^	NE	NE	NE	NE	NE	NE	LDL, total lipid, cholesterol, MDA, CRP, and triglycerides are elevated and HDL levels decreased in RA compared with control group.

Stamp et al., 2012 [20]	NE	PC: ^↑^p; CT: sf	NE	ALLA: ^↑^p	MPO: ^↑^p^#^^↑^sf^Δ^	NE	NE	MPO protein concentration is elevated in RA and promotes oxidative stress through the production of hypochlorous acid. There is a significant relationship between plasma MPO concentration and DAS-28.

Staroń et al., 2012 [21]	TBARS: ^↑^e	NE	NE	NE	CAT: ^*∗*^e; SOD: ^↓^e; GPx: ^*∗*^e	GSH: ^↓^e; -SH: ^↓^e	NE	There are no significant differences in CAT and GPx activities. SOD activity is lower and lipid peroxidation is increased in RA. GSH and -SH groups are significantly lower in RA.

Alver et al., 2011 [31]	MDA: ^↑^s, ^↑^e	NE	NE	NE	CAT: ^*∗*^e; SOD: ^↓^e; GPx: ^↓^e	GSH: ^↑^e	NE	The CAII autoantibody titers were significantly higher in RA. The increased erythrocyte oxidative stress in RA may be effective in the mechanism of CA II autoantibody production.

Aryaeian et al., 2011 [34]	MDA: ^↑^s	NE	NE	NE	GR: ^↓^e; AE: ^*∗*^s	*β*-C: ^↓^p; VE: ^↓^p	NE	There is an increased oxidative stress (MDA elevated) and a low antioxidant status (vitamin E, *β*-carotene, and GR activity diminished) in patients with RA.

Ediz et al., 2011 [25]	MDA: ^*∗*^wb, ^*∗*^s, ^↑^sf	NE	NE	NE	CAT: ^*∗*^wb, ^*∗*^s, ^*∗*^sf; GPx: ^*∗*^wb, ^*∗*^s, ^*∗*^sf; MPO: ^*∗*^wb, ^*∗*^s, ^↑^sf	NE	NE	There was positive correlation between ACPA levels and synovial MDA and MPO in ACCP (+) group. ACPA positivity seems to be associated with increased synovial fluid oxidant activity in RA.

Hassan et al., 2011 [10]	MDA: ^↑^s	NE	NE	NE	GPx: ^↓^e	GSH: ^↓^e	NE	Oxidative stress was increased in RA. DAS-28 significantly correlated with MDA levels and negatively with GSH.

Karaman et al., 2011 [30]	MDA: ^↑^p	NE	DNA sb: ^↑^L; MN: ^↑^L	NE	SOD: ^↓^p; GPx: ^↓^p	NE	NE	Elevated degree of oxidative stress in RA patients associated with DNA damage.

Shah et al., 2011 [32]	MDA: ^↑^p	NE	NE	NE	CAT: ^↓^p; SOD: ^↓^p; GPx: ^*∗*^p	GSH: ^↓^p	NE	Elevated ROS production disturbs redox status and can modulate the expression of inflammatory chemokines leading to inflammatory processes, exacerbating inflammation, and affecting tissue damage.

Desai et al., 2010 [22]	MDA: ^↑^wb	NE	NE	NE	SOD: ^↓^e; GR: ^↓^e	NE	NE	There is an increased oxidative stress and a decreased antioxidant defense in patients with RA.

Rho et al., 2010 [23]	F2-I: ^↑^U	NE	NE	NE	NE	NE	NE	F2-isoprostanes were higher in RA and they significantly modified the protective effect of HDL cholesterol against coronary calcification.

Tetik et al., 2010 [24]	NE	PC: ^↑^p	NE	NE	NE	-SH: ^↓^p	NE	Protein carbonyls content was higher in RA as compared to controls while the plasma -SH levels in RA was significantly lower than control. CRP levels were higher in RA.

ACPA: anti-citrullinated protein antibodies, ADMA: asymmetric dimethylarginine, AE: arylesterase, ALLA: allantoin, Anti-cap: total antioxidant capacity, AOPP: advanced oxidation protein products, CAII: carbonic anhydrase II autoantibody, CAT: catalase, CRP: C-Reactive Protein, CT: 3-chlorotyrosine, DAS-28: disease activity score, DNA sb: DNA strand breaks, e: erythrocyte, F2-I: F2-isoprostane, GPx: glutathione peroxidase, GR: glutathione reductase, GSH: reduced glutathione, GSSG: oxidized glutathione, H_2_O_2_: hydrogen peroxide, HDL: high density lipoprotein, IL: interleukin, L: lymphocytes, LDL: low density lipoprotein, MAA: malondialdehyde-acetaldehyde, MDA: malondialdehyde, MN: micronucleus, MPO: myeloperoxidase, n-b: neutrophils isolated from blood, n-sf: neutrophils isolated from synovial fluid, NADPH ox: nicotinamide adenine dinucleotide phosphate oxidase, NE: not evaluated, NO^∙^: nitric oxide, O_2_^−∙^: superoxide anion radical, OA: osteoarthritis, ^∙^OH: hydroxyl radical, p: plasma, PC: protein carbonyls, RA: rheumatoid arthritis, ROM: reactive oxygen metabolites, ROS: reactive oxygen species, RSNO: S-nitrosothiols, s: serum, -SH: thiol group, sf: synovial fluid, st: synovial tissue, SOD: superoxide dismutase, TBARS: thiobarbituric acid reactive substances, U: urine, VE: vitamin E, wb: whole blood, and *β*-C: -carotene.

^↑^Significantly elevated levels, ^↓^significantly diminished levels, ^*∗*^no significantly difference, and ^*❖*^marker measured with no comparison control group; ^#^protein concentration (no activity); ^Δ^significantly elevated levels of MPO protein concentration in SF compared with plasma (both derived from RA patients); ^✪^marker compared with neutrophils from RA patients.
